# Supplementary material for: Controlling Brain State Prior to Stimulation of Parietal Cortex Prevents Deterioration of Sustained Attention
Source: Cereb Cortex Commun. 2020 Sep 30;1(1):tgaa069. doi: 10.1093/texcom/tgaa069 (PMC8152938; doi:10.1093/texcom/tgaa069)
Supplement: EdwardsEtAl_SupportingInformation_Final_tgaa069 [file edwardsetal_supportinginformation_final_tgaa069.pdf]

Supplemental Information

Controlling brain state prior to stimulation of parietal cortex prevents deterioration of sustained attention

Grace Edwards[1,2], Federica Contò[1,3], Loryn K. Bucci[4], Lorella Battelli[1,2,5]

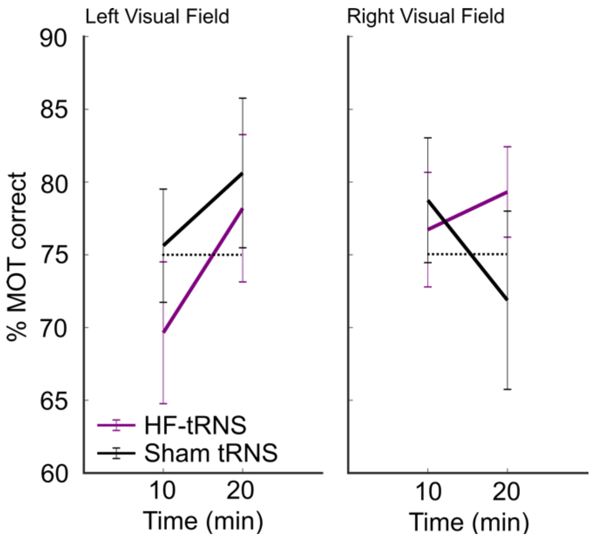

Supplemental Figure 1: Percentage correct MOT performance during HF-tRNS and Sham-tRNS in Experiment 1. Data split into left and right visual field, and first and last 10 minutes of stimulation.

	χ^2	DF	p-val
Main Effect: Stimulation	0.0637	1	0.801
Main Effect: Time	1.0276	1	0.311
Main Effect: Visual field	0.0361	1	0.849
Interaction: Stim x Time	1.7180	1	0.190
Interaction: Stim x VF	1.6742	1	0.196
Interaction: Time x VF	3.4154	1	0.065
Interaction: Stim x Time x VF	0.5063	1	0.477

Supplemental Table 1: Chi-square and p-val output of GLMER comparing performance *during* HF-tRNS and Sham-tRNS in Experiment 1.

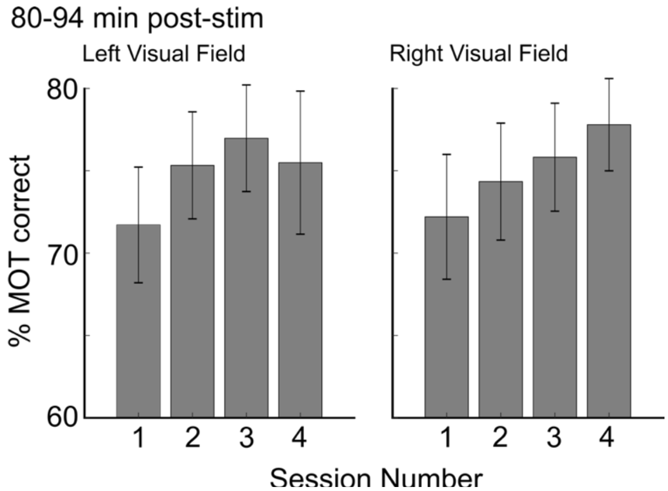

Supplemental Figure 2: Impact of multiple session MOT on sustained attention in Experiment 2. Percentage multiple object tracking accuracy change across session in the 80-94 minute time-bin post-stimulation.

<u>Model Comparisons</u>

Model comparisons were conducted on various *glmer* models to determine the best model fit for the data collected from experiment 1 and experiment 2.

Model Comparisons – Experiment 1

Syntax:

First we compare our hypothesized model of best fit (Mod2 – interaction between Stimulation and Time) with a simpler model (Mod1 – no interaction):

```
Mod1 <- glmer (Accuracy ~ Stimulation + Times + VF + (1|Subs), data = Multimethod, family = binomial)
Mod2 <- glmer (Accuracy ~ Stimulation * Times + VF + (1|Subs), data = Multimethod, family = binomial)

anova(Mod1,Mod2)
```

Mod	Df	AIC	BIC	loglik	deviance	Chisq	Chi Df	Pr(>Chisq)
Mod1	9	10411	10475	-5196.5	10393			
Mod2	14	10385	10485	-5178.6	10357	35.866	5	<0.000001

This demonstrates Mod2 is a better fit for our data than Mod1.

Next we add a level of complexity by determining if the additional variable of visual field in the interaction increases the fit of the model to our data:

```
Mod5 <- glmer (Accuracy ~ Stimulation * Times * VF + (1|Subs), data = Multimethod, family = binomial)

anova(Mod2,Mod5)
```

Mod	Df	AIC	BIC	loglik	deviance	Chisq	Chi Df	Pr(>Chisq)
Mod2	14	10385	10485	-5178.6	10357			
Mod5	25	10398	10576	-5173.9	10348	9.2764	11	0.5964

The lack of significant difference between Mod5 and Mod2 indicates that the additional complexity of Mod5 did not improve the fit to the data, therefore we reject Mod5 in favor of Mod2.

We also present Mod3 and Mod4 with interaction terms which also did not fit the data better than the simplest model, Mod1:

Mod3 <- glmer (Accuracy ~ Stimulation + Times * VF + (1|Subs), data = Multimethod, family = binomial)

anova(Mod1,Mod3)

Mod	Df	AIC	BIC	loglik	deviance	Chisq	Chi Df	Pr(>Chisq)
Mod1	9	10411	10475	-5196.5	10393			
Mod3	14	10418	10518	-5195.2	10390	2.6514	5	0.735

Mod4 <- glmer (Accuracy ~ Times + Stimulation * VF + (1|Subs), data = Multimethod, family = binomial)

anova(Mod1,Mod4)

Mod	Df	AIC	BIC	loglik	deviance	Chisq	Chi Df	Pr(>Chisq)
Mod1	9	10411	10475	-5196.5	10393			
Mod4	10	10410	10481	-5.195.1	10390	2.7434	1	0.09766

Model Comparisons – Experiment 2

Syntax:

We find the model which best fits our data is the model including the three way interaction between Stimulation x Time x Visual Field:

Mod1 <- glmer (Accuracy ~ Stimulation + Times + VF + (1|Subs), data = Multimethod, family = binomial)
Mod5 <- glmer (Accuracy ~ Stimulation * Times * VF + (1|Subs), data = Multimethod, family = binomial)

Mod	Df	AIC	BIC	loglik	deviance	Chisq	Chi Df	Pr(>Chisq)
Mod1	11	31465	31556	-15722	31443			
Mod5	49	31485	31891	-15694	31387	56.31	38	0.02817

No simpler model fit the data significantly:

Mod2 <- glmer (Accuracy ~ Stimulation * Times + VF + (1|Subs), data = Multimethod, family = binomial)

anova(Mod1,Mod2)

Mod	Df	AIC	BIC	loglik	deviance	Chisq	Chi Df	Pr(>Chisq)
Mod1	11	31465	31556	-15722	31443			
Mod2	26	31478	31694	-15713	31426	17.125	15	0.3115

Mod3 <- glmer (Accuracy ~ Stimulation + Times * VF + (1|Subs), data = Multimethod, family = binomial)

anova(Mod1,Mod3)

Mod	Df	AIC	BIC	loglik	deviance	Chisq	Chi Df	Pr(>Chisq)
Mod1	11	31465	31556	-15722	31443			
Mod3	16	31465	31597	-15716	31443	10.514	5	0.06192

Mod4 <- glmer (Accuracy ~ Times + Stimulation * VF + (1|Subs), data = Multimethod, family = binomial)

anova(Mod1,Mod4)

Mod	Df	AIC	BIC	loglik	deviance	Chisq	Chi Df	Pr(>Chisq)
Mod1	11	31465	31556	-15722	31443			
Mod4	14	31469	31585	-15721	31441	2.2816	3	0.516

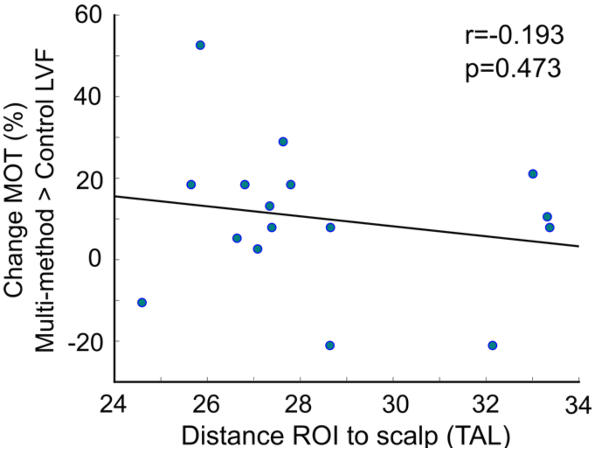

Supplemental figure 3: No significant correlation between stimulation site distance from scalp and percentage change in multiple object tracking accuracy in left visual field after multi-method stimulation.
